# Editors’ Overview: Experiments, Ethics, and New Technologies

**DOI:** 10.1007/s11948-015-9748-8

**Published:** 2016-01-09

**Authors:** Neelke Doorn, Shannon Spruit, Zoë Robaey

**Affiliations:** Department of Technology, Policy and Management, Delft University of Technology, PO Box 5015, 2600 GA Delft, The Netherlands

## Introduction

Technology has always been a vital component of human development, most often aimed at the advancement of human well-being. However, the introduction of new technologies may also introduce harmful consequences. Indeed, we often do not know beforehand the full spectrum of potential impacts and hazards that these new technologies may bring about. Conventional approaches to risk governance are not directly applicable to these fields due to high levels of uncertainty and ignorance. The inadequacies of these conventional approaches do not, however, warrant categorically rejecting the introduction of new technologies. At the same time, since those uncertain or unknown technological risks and dangers often only materialize after a long time (e.g., asbestos, DDT), there seems to be a need for flexible approaches that are adaptive to new information about the impact of technologies. A broad scholarship has been reflecting on how to deal with new and potentially risky technologies. Within that scholarship, some authors have argued to conceive of new technologies as social experiments and to look for the conditions under which such experiments are morally justified. In this special issue we look at the implications of using this social experiment lens under four overarching themes: philosophical underpinnings of the notion of experiments, the conditions that justify experimentation, the opportunities of experimentation, and how to define agents’ moral responsibility in the social experiment.

## Conceptual Overview

The first theme is about the philosophical underpinnings and conceptualization of the notion of experiment. The first contribution is by Hansson (2015), who emphasizes the need to distinguish between experiments, in the standard scientific sense of the term, and non-experimental observations. These are tailored to different epistemic needs. Hansson makes the distinction between action-guiding and epistemic experiments and reflects on the justification of these different types of experiments. Similarly, Kroes (2015) discusses the differences between experiments as used in the natural and social sciences and the notion of socio-technical experiments in the light of control and the possibility of intervention. Especially the notion of control, as commonly used in scientific experiments, will lose its standard meaning if applied to socio-technical systems, Kroes argues. Schiaffonati (2015) elaborates the notion of control in computer experiments. She introduces a distinction between a priori and a posteriori control. Whereas a priori control relies on anticipation, in a posteriori control the idea of full anticipation has been abandoned and control will be carried out after the artefact has been inserted into society.

This immediately relates to the second theme: under what conditions is social experimentation justified from an ethical point of view? Experimentation is often used in a pejorative sense (e.g. when the experimental subject is considered a “guinea pig”), which prompts the question how we can distinguish *responsible* from *irresponsible* experimentation. Van de Poel (2015) develops a framework for assessing the acceptability of such experiments based on the bioethical principles for experiments with human subjects: non-maleficence, beneficence, respect for autonomy, and justice. These four broad criteria can be further specified into a set of fifteen conditions, which are to be seen as prima facie moral obligations that are open to further specification for specific technologies and to revision in the light of new experiences. Acknowledging the experimental character of technological innovation, Schröder (2015) develops an account of socio-technical experiments that combines the experimental lens with insights from Actor-Network-Theory. Schröder’s framework includes both the epistemological realm of experiments (the “method”) but also the natural and material realm (the “things”) and the social realm (the delegation of “action” to technology). It could be used as a “sensitizing concept” to explore under what conditions experimentation is indeed responsible. She elaborates this framework on the basis of the geological disposal of nuclear waste, which is also the focal technology of Jan Bergen’s contribution (Bergen 2015). Bergen emphasizes the reversibility of consequences as a *condition sine qua non* for responsible experimentation. Responsible experimentation requires that we should be able to stop the experiment and undo its consequences when we think it is no longer desirable to proceed. He illustrates his argument on the basis of the geological disposal of nuclear waste. Kendig (2015) focuses on the early research phase of technology development and she shows how epistemic categories of cutting edge research into technologies prompt new ethical categories. Using the notion of proof of concept research she explains how the epistemic lens, through which we look at new technologies, influences how we interact with these technologies in a moral sense and which moral questions we should ask about these technologies. She develops the idea of extended agency ethics as an agent-based framework for addressing ethical challenges in proof of concept research. Recognizing that the locus of normative agency and intentionality in proof of concept research is distributed across the activities of different research groups and actors, this approach provides a naturalistic alternative for traditional individualistic approaches like consequentialism and deontology that are less suitable for dealing with technologies that are still in an experimental stage. The notion of responsible experimentation is also discussed in the contribution by Doorn (2015). Doorn discusses a case study in the water domain to explore the questions of when and under what conditions governance experiments are likely to be responsible experiments. She shows that governance experiments can be responsible experiments, but that effort should be put in how to organize these experiments and how to involve the stakeholders to ensure that these experiments do not come at the expense of legitimacy.

The third theme is about the opportunities of conceiving of technologies as social experiments. Is it applicable to all technologies or maybe only to some technologies? Or has it maybe even wider applicability? Hawkins (2015) applies the experimental lens to genetically modified organisms (GMOs). For Ronnie Hawkins, the experimental lens provides an alternative to the precautionary principle, which she deems unsuitable for evaluating complex technologies like GMOs. Hawkins’ main criticism to the precautionary approach is that it does not challenge the underlying economic assumptions, like discounting the future and trade-offs between environmental regulation and risks that come with regulation. Instead, Hawkins defends an experimental approach, which she links to the ecological notion of resilience. Asveld (2016) discusses the lock-in that has occurred in the policies of the European Union concerning the development of first generation biofuels. Initially hailed as a green sustainable technology, these first generation biofuels have become controversial due to uncertainties about their physical impact, their moral evaluation, and their institutional embedding. Since a considerable number of member states developed an economic interest in these first generation biofuels, alteration of the EU policies to accommodate for these effects met with fierce resistance. Asveld shows how an experimental approach to the development of sustainable bio-based technologies may have prevented such a lock-in. Pieters et al. (2015) apply the experimentation paradigm to the context of cyber-security. Whereas the new technologies as social experiments paradigm is primarily discussed in the context of safety, Pieters and colleagues show how insights from the security domain may provide additional conditions for responsible experimentation to make it applicable for situations in which the primary threats come from adversarial use of the technology and deliberate attacks rather than a lack of safety of the technology itself. Like Pieters and colleagues, Stilgoe (2015) also tries to move beyond mere direct safety risks. Stilgoe applies the experimental lens to geoengineering. Since the outcomes of geoengineering are highly speculative, geoengineering seems to be the paradigmatic case of a social experiment. On the basis of the first UK field geoengineering test site, Stilgoe shows how the involvement of social scientists in this project lead to a renegotiation of what is known and what is unknown and also to the inclusion of more than just the direct risks. This new mode of governance, which he refers to as collective experimentation, allows us to “experiment with experimentation,” therewith extending the scope of experimentation beyond technology itself. According to Stilgoe, experiment should be seen as a verb rather than a noun.

This brings us to the last theme in this special issue, which relates to the notion of responsibility in a social experiment. During the development of a technology and after it has been introduced in society several actors become involved in the experiment. This may blur the distribution of responsibilities between different parties involved and prompt questions as to what these responsibilities entail. It thereby also links to issues of democratization of science. Spruit et al. (2015) discuss the topic of responsibility in relation to nanoscale science and engineering. Since a well-defined nano-community is lacking, the attribution of responsibility in the development of nanotechnologies is problematic. Spruit and colleagues argue that if we want responsible development in dispersed scientific and engineering fields, like nanotechnology, individual researchers have the duty to organize themselves to create collective agents that have the capacity to steer technological development. The same issue of ascribing responsibility is in the context contamination of GMOs. Robaey (2016) argues that since owners reap benefits off of new technologies, they should have forward looking responsibilities. Also, the lack of knowledge about those technologies does not remove moral responsibility on the effects of the GMOs they own. Using the lens of social experimentation allows defining the forward-looking moral responsibility of owners as a set of epistemic virtues they should strive to develop in order to learn and react to the technologies they are using and spreading. Krabbenborg (2015) focuses on the involvement of civil society actors as knowledgeable dialogue partner in the development and governance of emerging technologies. Based on Dewey’s notion of reflective inquiry, Krabbenborg argues that scientists and engineers working on new and emerging technologies have a responsibility to participate in such reflective inquiries with the publics. In order to facilitate this, existing institutions have to evolve as well to allow inquiry and deliberation among different relevant actors. Like Stilgoe, Krabbenborg argues for flexibility to allow for tentative approaches rather than strict arrangements. With this special issue, we hope to have provided insights on how an experimental lens may provide new ways to reflect on the moral evaluation of new technologies. We thank all the authors for their valuable contribution.
